# Rheolytic Thrombectomy with or without Adjunctive Indwelling Pharmacolysis in Patients Presenting with Acute Pulmonary Embolism Presenting with Right Heart Strain and/or Pulseless Electrical Activity

**DOI:** 10.1155/2011/246410

**Published:** 2011-12-28

**Authors:** J. Hubbard, W. E. A. Saad, S. S. Sabri, U. C. Turba, J. F. Angle, A. W. Park, A. H. Matsumoto

**Affiliations:** ^1^Department of Radiology, University of Virginia Health System, Charlottesville, VA 22908, USA; ^2^Division of Interventional Radiology, University of Virginia Health System, Charlottesville, VA 22908, USA

## Abstract

*Purpose*. To evaluate the safety and efficacy of the Possis rheolytic thrombectomy with or without indwelling catheter-directed pharmacolysis for the treatment of massive pulmonary embolus in patients presenting with right heart strain and/or a pulseless electrical activity (PEA). *Materials and Methods*. Retrospective review of patients undergoing pulmonary pharmacolysis was performed (07/2004–06/2009). Pre- and posttreatment Miller index scoring weres calculated and compared. Patients were evaluated for tPA doses, ICU stay, hospital stay, and survival by Kaplan-Meier analysis. *Results*. 11 patients with massive PE were found, with 10/11 presenting with a Miller score of >17 (range: 16–27, mean: 23.2). CTPA and/or echocardiographic evidence of right heart strain was found in 10/11 patients. 3 (27%) patients presented with a PEA event. Two (18%) patients had a contraindication to pharmacolysis and were treated with mechanical thrombectomy alone. The intraprocedural mortality was 9% (*n* = 1/11). Of the 10 patients who survived the initial treatment, 7 patients underwent standard mechanical thrombectomy initially, while 5 received power pulse spray mechanical thrombectomy. Eight of these 10 patients underwent adjunctive indwelling catheter-directed thrombolysis. The mean catheter-directed infusion duration was 18 hours (range of 12–26 hours). The average intraprocedural, infusion, and total doses of tPA were 7 mg, 19.7 mg, and 26.7 mg, respectively. There was a 91% (10/11) technical success rate. The failure was the single mortality. Average reduction in Miller score was 9.5 or 41% (*P* = 0.009), obstructive index of 6.4 or 47% (*P* = 0.03), and perfusion index of 2.7 or 28% (*P* = 0.05). Average ICU and hospital stay were 7.4 days (range 2–27 days) and 21.3 days (range 6–60 days), respectively. Intent to treat survival was 90% at 6, 12, and 18 months. *Conclusion*. Rheolytic thrombectomy with or without adjunctive catheter-directed thrombolysis provides a safe and effective method for treatment of acute PE in patients who present with right heart strain and/or a PEA event.

## 1. Introduction

Pulmonary embolism (PE) is a common event resulting in an estimated 150,000 deaths per year in the United States, with an apparent increasing frequency as the sensitivity of diagnostic imaging improves [1–3]. While the majority of these patients may be minimally symptomatic and can be treated medically with anticoagulation alone, patients with underlying cardiopulmonary disease and those patients who show right ventricular compromise or hemodynamic instability may require acute intervention to decrease clot burden [1, 2, 4]. In patients who present with compromised hemodynamics or cardiac function, a variety of treatment options have been utilized to treat acute PE, including surgical embolectomy, intravenous fibrinolysis, catheter-directed pharmacolysis, and transcatheter mechanical clot fragmentation with or without thrombectomy [2, 5–20].

The general aim of catheter-directed therapy in the setting of hemodynamically compromised patients with acute PE is to debulk and/or redistribute the obstructive clot, rendering it less hemodynamically significant [4]. Most often, mechanical thrombectomy is used alone, and adjunctive use of indwelling transcatheter-directed thrombolytic infusions is rarely reported [2]. Numerous catheter-directed mechanical thrombectomy devices have been utilized for acute PE, including the AngioJet device (Xpeedior Possis Medical, Minneapolis, Minn) [2, 4–10]. The utilization of the AngioJet device in the setting of acute PE has been a controversial topic [2, 4–10]. Prior studies have shown that the AngioJet can be highly effective in reducing clot burden [2], however adverse events such as significant bradycardia and even asystole have been reported [1, 4–10].

The purpose of this study is to evaluate the safety, effectiveness, and long-term outcome of rheolytic thrombectomy using the Angiojet Possis device with or without the supplemental use of catheter-directed thrombolysis in patients with acute pulmonary emboli who present with right heart strain and/or pulseless electrical activity.

## 2. Materials and Methods

### 2.1. Patient Population

A retrospective analysis was performed of all consecutive patients treated for acute pulmonary embolus (PE) by endovascular means from July 2004 to June 2009. Internal Review Board permission for the study was obtained based upon the secondary use of anonymous data. Patients with tumor embolus were excluded from the study. Catheter-directed techniques for management of acute PE were utilized in patients in whom there was a large clot burden and evidence of hemodynamic instability and/or right heart strain. Patients were noted for their age and gender. Pulmonary embolism was diagnosed by a contrast-enhanced computed tomography pulmonary arteriogram (CTPA). Echocardiography was also performed in several patients at the discretion of the referring physician to evaluate right heart function.

### 2.2. Definitions

Massive PE was classified by CTPA criteria as having a Miller score of >17 and/or CTPA and/or echocardiographic evidence of right heart strain [21]. The Miller score is a sum of two components: (1) obstructive index and (2) perfusion index [21]. Right ventricular heart strain by CTPA was defined as paradoxical bowing of the interventricular septum into the left ventricle during systole and right ventricular dilatation, with or without dilatation of the inferior vena cava, the hepatic veins, the coronary sinus, and the azygous vein [22]. Criteria for right heart strain on echocardiography include increased right ventricular size, paradoxical interventricular septal contraction during systole, and increased pulmonary artery pressures [22, 23].

### 2.3. Technique

The desired endpoint of catheter-directed therapy was to debulk (remove or dissolve) thrombus or redistribute the hemodynamically significant clot from the central pulmonary arteries to the more peripheral branches to relieve the afterload (strain) on the right ventricle [4]. The intention for all patients was to undergo catheter-directed mechanical thrombectomy debulking (with or without intraprocedural pharmacolysis). When there was no contraindication to thrombolysis, mechanical thrombectomy was followed by at least 12 hours of indwelling catheter-directed pharmacolysis and a subsequent “second look” pulmonary angiogram. If there was a contraindication to pharmacolysis or if signs of bleeding developed during intraprocedural administration of a bolus of tissue plasminogen activator (tPA, Alteplase, Genetech, South San Francisco, Calif), the patients underwent mechanical thrombectomy without adjunctive indwelling catheter-directed pharmacolysis.

Pulmonary angiograms were performed using standard angiographic techniques via a transfemoral approach unless there was chronic inferior vena cava thrombus. In these instances, a basilic vein approach was chosen for catheter access. The catheter utilized to catheterize the pulmonary artery was a 7-French angled pigtail catheter (Montefiori, -Cook, Inc, Bloomington, Ind). Mechanical thrombectomy was performed utilizing the over-the-wire Possis Angiojet. The Angiojet has a traditional suction rheolytic mode, but it can be used in a power pulse-spray mode [24]. Either the standard, power pulse spray, or both methods were used during mechanical thrombectomy, depending on the operator's discretion.

The traditional suction rheolytic mode of the AngioJet relies on retrograde injection of high-pressure saline jets to create a low-pressure zone around the openings near the catheter tip (Bernoulli principle). The recirculating heparinized saline creates a vortex (Venturi effect) causing fragmentation of the thrombus into smaller fragments. The injected saline and fragmented thrombus are removed via the suction lumen of the device resulting in an euvolemic state while debulking, removing, and redistribution of the emboli. The saline used can be mixed with heparin or tPA. The power pulse-spray mode is when the suction lumen of the AngioJet device is blocked with a stopcock, which generates a powerful spray when the device is activated. This spray will not only mechanically fragment clot, but also deliver tPA into the interstices of the clot and potentially activate more clot-bound plasminogen to effect more rapid pharmacolysis of the clot [24].

When no contraindication to or active bleeding developed after intraprocedural tPA administration existed, postprocedural indwelling catheter-directed tPA thrombolysis with tPA was performed. The catheters utilized for tPA administration were either 5-French infusion multisidehole catheters with 10-centimeter infusion lengths or 5-French diagnostic pigtail catheters. After indwelling transcatheter pharmacolysis, surviving patients were returned for a completion pulmonary angiogram and discontinuation of the infusion catheters.

### 2.4. Study Endpoints and Followup

Thrombus burden was calculated for all patients using the obstructive index (Number of segmental arteries involved-OI), perfusion index (Relative blood flow to upper, mid and lower lung zones-PI), and Miller index score (Summation of OI and PI) preprocedurally using both CTPA and angiographic images and rescored based upon the posttreatment angiograms [21].

Technical success was defined as a decrease in clot burden as determined on the immediate posttreatment angiogram. Minor complications included bleeding not requiring blood transfusion, transient renal dysfunction not requiring the need for dialysis, and/or catheter induced arrhythmias-not requiring treatment. Major complications were defined as bleeding requiring a blood transfusion, intracranial hemorrhage, death, arrhythmia requiring treatment, renal failure requiring hemodialysis, and/or the need for prolonged hospitalization as a direct result of the treatment [4].

Postprocedure patients were followed, noting the duration of intensive care unit stay and the number of days of hospitalization. After discharge from the hospital, the patient records were evaluated for clinic visits, complaints, and readmission. Full recovery was defined as ambulatory hospital discharge to home without cardiopulmonary sequela (i.e., no need for home oxygen requirements) or the need for skilled nursing care.

### 2.5. Statistical Analysis

The preprocedural and postprocedural obstructive index, perfusion index, and Miller score in all patients were compared using Student's *t*-test analysis. A *P* value of <0.05 was considered statistically significant. Intent to treat patient survival was determined by the Kaplan-Meier method and expressed as a percentage with a 95% confidence interval (95% CI).

## 3. Results

Between 2004 and 2009 (6 years), 12 consecutive patients with acute PE presented with imaging evidence of right ventricular strain, hemodynamic instability, or a PEA event were treated with rheolytic mechanical thrombectomy with or without adjunctive catheter-directed tPA infusion into the pulmonary artery clot. One patient was excluded from analysis for having intraluminal tumor thrombus as the cause for PE. The mean age of the remaining 11 patients was 60.2 years (range: 15–75 years). Nine patients were male and 2 female. Three patients presented with a PEA event (3/11, 27%). Six of the patients were evaluated with both CTPA and echocardiography, 3 patients had echocardiography only, and 2 had CTPA only. Details of the indications of PE fibrinolysis are in Table 1.

One of the eleven patients had a contraindication to tPA treatment (intracranial neoplasm) (Patient no.5, Table 1), and 1 patient died prior to beginning of the intraprocedural thrombolysis (Patient no.11, Table 1). A third patient exhibited epistaxis during the intraprocedural administration of tPA and was not treated with indwelling catheter-directed pharmacolysis after the initial procedure.

**Figure 1:**
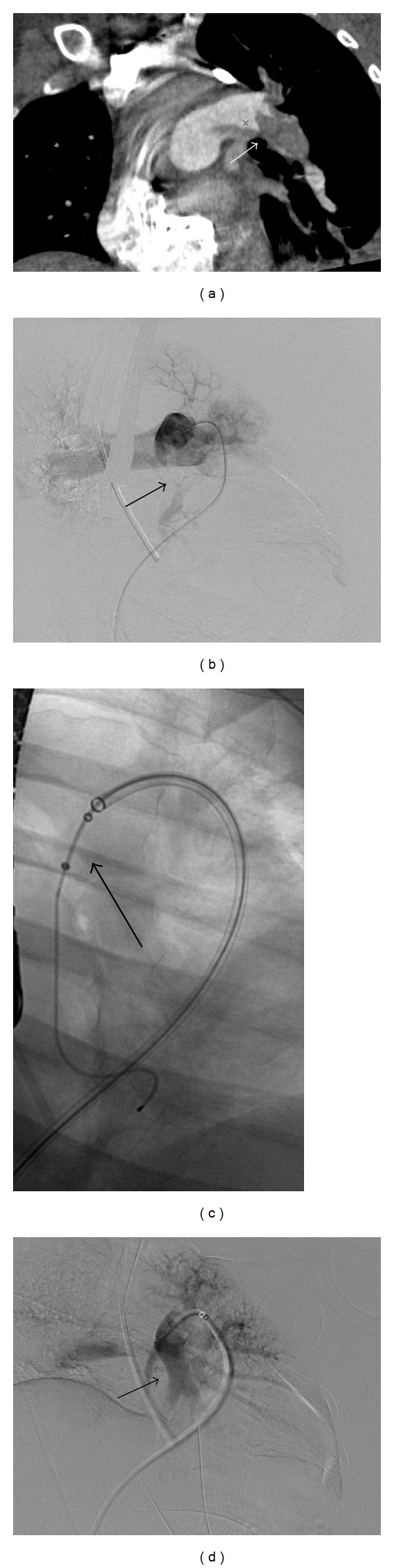
This is 44-yr-old woman with acute chest pain shortness of breath and tachycardia. Oblique coronal CT image (a) and pulmonary angiogram (b) showed massive left main and lower lobe PA embolus (arrows). Mean pulmonary artery pressure on initial study was 34 mm Hg. A 6-French Angiojet device (arrow) was used (c) followed by catheter-directed thrombolysis for 13 hours. Follow-up angiogram demonstrated significant decrease in clot burden (arrow) (d). Mean pulmonary artery pressure decreased from 34 to 18 mmHg.

Therefore, of the 10 patients treated to completion (and excluding the one patient who expired), 7 underwent standard rheolytic thrombectomy, while five were treated using the power pulse-spray technique using a tPA mixture only (the intraprocedure tPA dose ranged from 4–10 mg, mean 7 mg) during the initial procedure. The patient who died in the procedure, died prior to starting rheolytic therapy (mechanical thrombolysis). Eight of these 10 patients underwent adjunctive postprocedural indwelling catheter-directed pharmacolysis, Four of these 8 patients had the infusion administered via a unilateral infusion catheter (3 in the right pulmonary artery and 1 in the left). Two patients had bilateral infusion catheters placed, and one patient had an infusion catheter placed in the right pulmonary artery and the sheath advanced into the main pulmonary artery for tPA infusion. The patient with chronic thrombus within the IVC had an infusion catheter placed in the right pulmonary artery and another catheter was positioned in the IVC (See Figure 1). Technical details of the PE fibrinolysis procedure including the tPA doses and routes of administration are shown in Table 1.

The average indwelling transcatheter lysis dose was 19.7 mg of tPA, and the average total dose (intraprocedural and adjunctive infusion of tPA) was 26.7 mg tPA. Overnight infusion of tPA was performed at a total dose of 1.0 mg/hr, with the total dose infused being split between two catheters if bilateral infusion catheters were placed. Patients returned for a repeat pulmonary arteriogram the following day. Infusion time ranged from 12 to 53 hrs (average 23.6). Only one patient required more than 24 hours of lytic infusion, and this patient presented with acute on chronic PE. Five of the patients underwent intraprocedure inferior vena cava (IVC) filter placement. IVC filter placement was at operators discretion.

Technical success on a basis of an intent to treat was 91% (10/11, with the tumor thrombus patient excluded), with the one failure being the patient that expired during the procedure. Treatment effects on the Miller score and its two components are shown in Table 2. These numbers correlate to a 47% decrease in the degree of obstruction, a 28% decrease in the perfusion index, and 41% decrease in the overall Miller score (*P* = 0.009). The effect of treatment on pulmonary artery pressure demonstrates a trend toward decreased pressures following treatment. However, the results were not statistically significant due to the small subset of patients in whom pressures were measured pre- and posttreatment (*n* = 7), shown in Table 3.

Following treatment, the intensive care unit (ICU) and hospital stays were 7.4 days (range 2–27 days) and 21.3 days (range 6–60 days), respectively. Patients were followed for a mean of 17.1 months (range = 0.2–32 months). The survival on an intent to treat basis using the Kaplan Meier survival curve was 91% (10/11) at 6, 12, and 18 months (95% CI: 80–100%). All 10 surviving patients were functioning well without limitation and without further hospitalizations or oxygen requirement.

## 4. Discussion

The role of catheter-directed management of acute pulmonary embolus (PE) associated with hemodynamic instability and right ventricular dysfunction continues to evolve and has yet to be determined [4, 25]. However, The American College of Chest Physicians recommends that transcatheter thrombectomy not be used for the majority of patients with acute PE and that it should be reserved for highly compromised patients with massive PE and either no time for peripheral fibrinolysis/thrombolysis to work (acutely critical clinical setting) and/or contraindications to fibrinolysis/thrombolysis [4, 26]. As a result, transcatheter management of acute PE is mostly utilized in desperate clinical situations which are likely to be associated with a high likelihood of patient mortality.

However, the specific clinical application of transcatheter management of acute PE is still unclear, particularly with regards to what defines hemodynamic instability and what the prognostic criteria are that will help to predict which patient will benefit from catheter-directed therapy. The Society of Interventional Radiology Reporting Standards for Endovascular Treatment of Pulmonary Embolism identifies risk factors for predicting high mortality rates with acute PE. These risk factors could be used as relevant inclusion criteria for employing transcatheter techniques for the management of acute PE. Risk factors for poor outcome in patients with massive pulmonary embolus and potentially relevant inclusion criteria for transcatheter management of pulmonary embolus. The criteria are the following.

- Arterial hypotension < 90 mmHg,
- Circulatory collapse requiring cardiopulmonary resuscitation,
- Shock-causing peripheral hypotension and hypoperfusion,
- Right heart strain by echocardiography,
- Significant pulmonary embolus with contraindication to anticoagulation,
- Widened arterial-alveolar O_2_ gradient > 50 mmHg, [4, 27].

However, one can deduce from this that there should be careful selective criteria, especially given that the unstable patient after an acute PE probably has a mortality rate of 10% immediately and 30% within 30 days [26–31]. In addition, patients with acute PE and hemodynamic instability have an associated 3- to 7-fold increase in mortality, with the majority of these deaths occurring within an hour of presentation [32]. Another significant variable that has affected the establishment of standard guidelines for use of catheter-directed therapy for acute PE is the large variety of mechanical thrombectomy devices and/or techniques utilized for acute PE; even the specific thrombolytic drug, doses, and methods of drug delivery have been highly variable over the years. In the 1990s, the use of Urokinase (abbokinase, Abbott Labs, Abbott Park, ILL) predominated within the United States market, and most operators were familiar with this drug. In the last decade, tPA has been the most widely used lytic in the United States market. In addition, there has been a plethora of mechanical thrombectomy devices and thrombolysis infusion catheters. This lack of uniformity in devices and drugs adds to the complexity of reporting on the experience with transcatheter treatment of acute PE.

The use of the Possis AngioJet for acute PE is a particularly controversial topic due to concerns of its safety in this vascular bed, particularly in a critically unstable situation [1, 4]. Furthermore, it is difficult to definitively ascertain, when reporting bradycardia and asystole within the procedure, whether these phenomena are from the Possis AngioJet itself, an effect of the red cell lysis and adenosine release, or from the underlying condition or a combination.

One of the theoretical advantages of combining transcatheter mechanical thrombectomy and pharmacolysis is the overall reduction in the lytic dose, the potential for reducing bleeding complications and the more rapid tempo of clot clearance, which is suggested in our small series. Previously published reports have demonstrated a bleeding rate of approximately 18% for patients with acute, massive PE treated with thrombolysis [33]. We had only one case (9%) of minor bleeding with no clinical sequelae in our series.

The major complications reported in the literature associated with the use of the rheolytic catheter for acute PE are bradyarrythmias and asystole [1, 4–10]. The cause of these complication is believed to be secondary to the hemolysis that occurs with this thrombectomy device and the subsequent release of adenosine [1, 4–10]. No significant bradyarrythmias were noted in our patients. However, operators were cognizant of the potential for bradycardia and activated the AngioJet for short bursts (usually less than 10 pulses at a time), allowing 20–30 seconds of time between activations to allow for clearance of compounds, such as adenosine, that might result during device activation. The one patient who expired became asystolic shortly into the procedure, but prior to initiation of any mechanical thrombectomy device or pharmacolysis. An autopsy was refused.

The primary aim of catheter-directed therapy (debulking and/or redistributing the central PE) was demonstrated by the impact of therapy on the obstructive index (OI) and the perfusion index (47% versus 28%, resp.). However, the larger effect on the OI as compared with the PI is not surprising given that the mechanical thrombectomy likely caused more fragmentation in the acute treatment setting than actual clot removal. Incomplete removal of these small fragmented pieces of emboli would result in decreased perfusion to peripheral pulmonary artery branches despite significant improvement in the central, obstructive portion of the clot. In theory, catheter infusion of tPA for at least 12 hours should continue to improve the perfusion index.

 Most patients in our series were limited to less than 1 week of time in the ICU, with the single outlier being the patient with acute on chronic thrombus. The patents also were discharged within 30 days of their diagnosis, again the only outlier being the patient with acute on chronic thrombus (who also had IVC occlusion). However, long term survival was excellent with 91% (10/11) of the patients (10/10 or 100% if the patient who expired prior to initiation of catheter-directed therapy is excluded from the survival data) who were alive and well at 18 months of followup. The mortality rate reported in the literature after mechanical thrombectomy and pharmaceutical thrombolysis utilizing the Possis AngioJet approximates 12% [2] which is similar to our experience. This survival data with percutaneous therapy is significantly better than compared to the overall population of patients with massive PE which approaches 70% at 30 days from presentation [2, 26–31].

The current study has significant limitations owing to its retrospective nature and its small sample size. These weaknesses are the result of careful patient selection for catheter-directed therapy. Studies of greater substance should involve multiple centers, preferably in a randomized control trial or at least in a registry with objective inclusion criteria and a standardized treatment algorithm.

In addition to these limitations and despite the small sample size, variations in the catheter-directed treatment techniques were seen within the study, particularly the use of the traditional Possis thrombectomy technique (7 cases) versus the power pulse Possis thrombectomy method (3 cases) due to operator preference rather than objective criteria. In addition, the Angiojet system provides multiple catheters, each calibrated to treat varying diameter vessels. In our series, the Xpeedior catheter, which is intended to treat up to a 12 mm diameter vessel was used since our personal experience has shown that the other catheters which are designed to use in larger vessels result in extensive hemolysis [34]. Other variations contributing to study limitations were in the pre- and postprocedural diagnostic imaging for PE load and signs of right heart strain. Furthermore, intra- and postprocedural pulmonary artery pressures and oxygen saturations were not obtained consistently to allow for acute quantitative analysis of the hemodynamic effects of the therapy.

In conclusion, patients with acute PE who present with evidence for right ventricular strain or an acute PEA event can be treated with percutaneous mechanical thrombectomy with the Possis Angiojet with or without adjunctive use of indwelling catheter-directed pharmacolysis relatively safely with very good early and mid-range survival outcomes.

## Figures and Tables

**Table 1:** Cardiopulmonary status and Possis Angiojet and technical details.

Patients	Cardioplumonary status			Possis Angiojet mode		tPA dose and administration route		
	Miller score	PEA/code	RV-strain	Pulse- spray	Aspiration	Intra-op tPA	Indwelling catheter tPA	Total tPA Dose
Patient #1	25	No	CTPA + Echo	No	Yes	4 mg	12 mg	16 mg
Patient #2	17	Yes	CTPA + Echo	No	Yes	8 mg	21 mg	29 mg
Patient #3	21	Yes	CTPA	Yes	Yes	8 mg	14 mg	22 mg
Patient #4	16	Yes	CTPA + Echo	Yes	Yes	6 mg	21 mg	27 mg
Patient #5*	26	No	CTPA + Echo	No	Yes	No	No	No
Patient #6	24	No	CTPA + Echo	Yes	No	10 mg	14 mg	24 mg
Patient #7	25	No	CTPA	Yes	Yes	8 mg	18 mg	26 mg
Patient #8	27	No	Echo	No	Yes + T	No	No**	No**
Patient #9	25	No	CTPA + Echo	Yes	No	8 mg	26 mg	34 mg
Patient #10	24	No	Echo	No	Yes	4 mg	31.5 mg	35.5 mg
Patient #11	25	No	Echo	No	Yes + T***	Patient Died		

*Patient had glioblastoma multiform (GBM) and was contraindicated to fibrinolysis.

**Patient had active epistaxis and did no undergo postprocedural catheter-directed fibrinolysis.

***The intention was to use aspiration Possis rheolytic, however, patient died on the table prior to its use. An Arrow-Trerotola PTD device was used (Arrow Intl. Inc. Reading, Pa).

+T: Arrow-Trerotola PTD device used (Arrow Intl. Inc. Reading, Pa).

**Table 2:** Preprocedural and posttreatment change in Miller score and its components.

	Preprocedure		Posttreatment		Mean nominal change in score	*P*-value
	Mean	Range	Mean	Range		
Obstructive index	13.5	9–16	7.1	1–13	6.4	0.03
Perfusion index	9.8	7–12	7.1	5–14	2.7	0.05
Miller score*	23	16–27	13.5	6–27	9.5	0.009

Miller score is the sum of the obstructive and the perfusion indices.

Reference: Miller et al. [21].

**Table 3:** Preprocedural and posttreatment pulmonary artery pressures (*n* = 7).

	Preprocedure		Posttreatment		Average change in pressure	*P*-value
	Mean	Range	Mean	Range		
PA pressure (mmHg)	38.6	32–45	28.7	15–53	7.9	0.07

PA: Pulmonary artery.

mmHg: Millimeters mercury.
